# Habitually Skipping Breakfast Is Associated with the Risk of Gastrointestinal Cancers: Evidence from the Kailuan Cohort Study

**DOI:** 10.1007/s11606-023-08094-7

**Published:** 2023-03-03

**Authors:** Tong Liu, Yiming Wang, Xiaomeng Wang, Chenan Liu, Qi Zhang, Mengmeng Song, Chunhua Song, Qingsong Zhang, Hanping Shi

**Affiliations:** 1 Department of Gastrointestinal Surgery/Clinical Nutrition, Capital Medical University Affiliated Beijing Shijitan Hospital, Beijing, 100038 China; 2 Beijing International Science and Technology Cooperation Base for Cancer Metabolism and Nutrition, Beijing, 100038 China; 3 Key Laboratory of Cancer FSMP for State Market Regulation, Beijing, 100038 China; 4 Department of Hepatobiliary Surgery, Kailuan General Hospital, Tangshan, 063000 China; 5 Department of Education, Capital Medical University Affiliated Beijing Shijitan Hospital, Beijing, 100038 China; 6 Department of Education, Peking University Ninth School of Clinical Medicine, Beijing, 100038 China; 7 Department of Epidemiology and Statistics, College of Public Health, Zhengzhou University, ZhengzhouHenan, 450001 China; 8 Department of General Surgery, Kailuan General Hospital, Tangshan, 063000 China

**Keywords:** Breakfast, Frequency, Gastrointestinal cancer, Prospective

## Abstract

**Background:**

Habitually skipping breakfast may promote the initiation and progression of gastrointestinal (GI) cancers, which have never been systematically explored in large-scale prospective studies.

**Methods:**

We prospectively examined the effects of breakfast frequency on the occurrence of GI cancers among 62,746 participants. The hazard ratios (HRs) and 95% confidence intervals (95% CIs) of GI cancers were calculated by Cox regression. The CAUSALMED procedure was used to perform the mediation analyses.

**Results:**

During a median follow-up of 5.61 (5.18 ~ 6.08) years, 369 incident GI cancer cases were identified. Participants who consumed 1–2 times breakfasts per week exhibited an increased risk of stomach (HR = 3.45, 95% CI: 1.06–11.20) and liver cancer (HR = 3.42, 95% CI: 1.22–9.53). Participants who did not eat breakfast had an elevated risk of esophageal (HR = 2.72, 95% CI: 1.05–7.03), colorectal (HR = 2.32, 95% CI: 1.34–4.01), liver (HR = 2.41, 95% CI: 1.23–4.71), gallbladder, and extrahepatic bile duct cancer (HR = 5.43, 95% CI: 1.34–21.93). In the mediation effect analyses, BMI, CRP, and TyG (fasting triglyceride-glucose) index did not mediate the association between breakfast frequency and the risk of GI cancer incidence (all *P* for mediation effect > 0.05).

**Conclusions:**

Habitually skipping breakfast was associated with a greater risk of GI cancers including esophageal, gastric, colorectal, liver, gallbladder, and extrahepatic bile duct cancer.

**Trial Registration:**

Kailuan study, ChiCTR–TNRC–11001489. Registered 24 August, 2011-Retrospectively registered, http://www.chictr.org.cn/showprojen.aspx?proj=8050

**Supplementary Information:**

The online version contains supplementary material available at 10.1007/s11606-023-08094-7.

## INTRODUCTION

Gastrointestinal (GI) cancers are costly and deadly. According to 2020 global cancer statistics,^1^ three of the top 5 cancer types resulting in death are GI cancers, including colorectal (9.4%), liver (8.3%), and stomach (7.7%) cancers. In China, stomach cancer is the second leading cause of cancer death, followed by liver, esophageal, and colorectal cancer.^2^ As one of the most replicative tissues in the body, the GI tract’s cells have a short lifespan. Physical, biological, and chemical damage continually affects the digestive system, increasing the chance of oncogenic alterations. Even though risk factors for GI malignancies differ depending on the site, there are still certain common risk factors, such as drinking,^3^ smoking,^4^ obesity,^5^ infectious agents,^6^ and chronic inflammation.^7^ Exploring the common risk factors for GI cancers and performing high-risk population screenings can help to prevent GI cancers.

Breakfast is commonly referred to as the most essential meal of the day and provides nutrients for the body after overnight fasting.^8^ A sizable amount of evidence on the benefits of breakfast focuses on health outcomes rather than nutritional results. In recent years, breakfast skipping has been proven to be associated with obesity,^9^ impaired glucose metabolism,^10^ cardiovascular disease,^11^ impaired cognitive function,^12^ and even cancer.^13^ Recently, a prospective study conducted in China found that habitually skipping breakfast is associated with chronic inflammation, as assessed by the C-reactive protein (CRP) concentration.^14^ As described previously, due to the close association between inflammation and GI cancers, the habitual breakfast skipping may result in the initiation and progression of malignant tumors in the digestive system. However, no prospective study has been conducted to explore this association.

The Kailuan study is an ongoing, longitudinal cohort study, and its purpose is to explore the risk factors for chronic diseases, including cancer. The assessment of breakfast frequency and the follow-up of incident cancer events offered us a great opportunity to explore whether breakfast frequency was associated with the risk of GI cancer incidence.

## METHODS

### Study Population

**Fig. 1:**
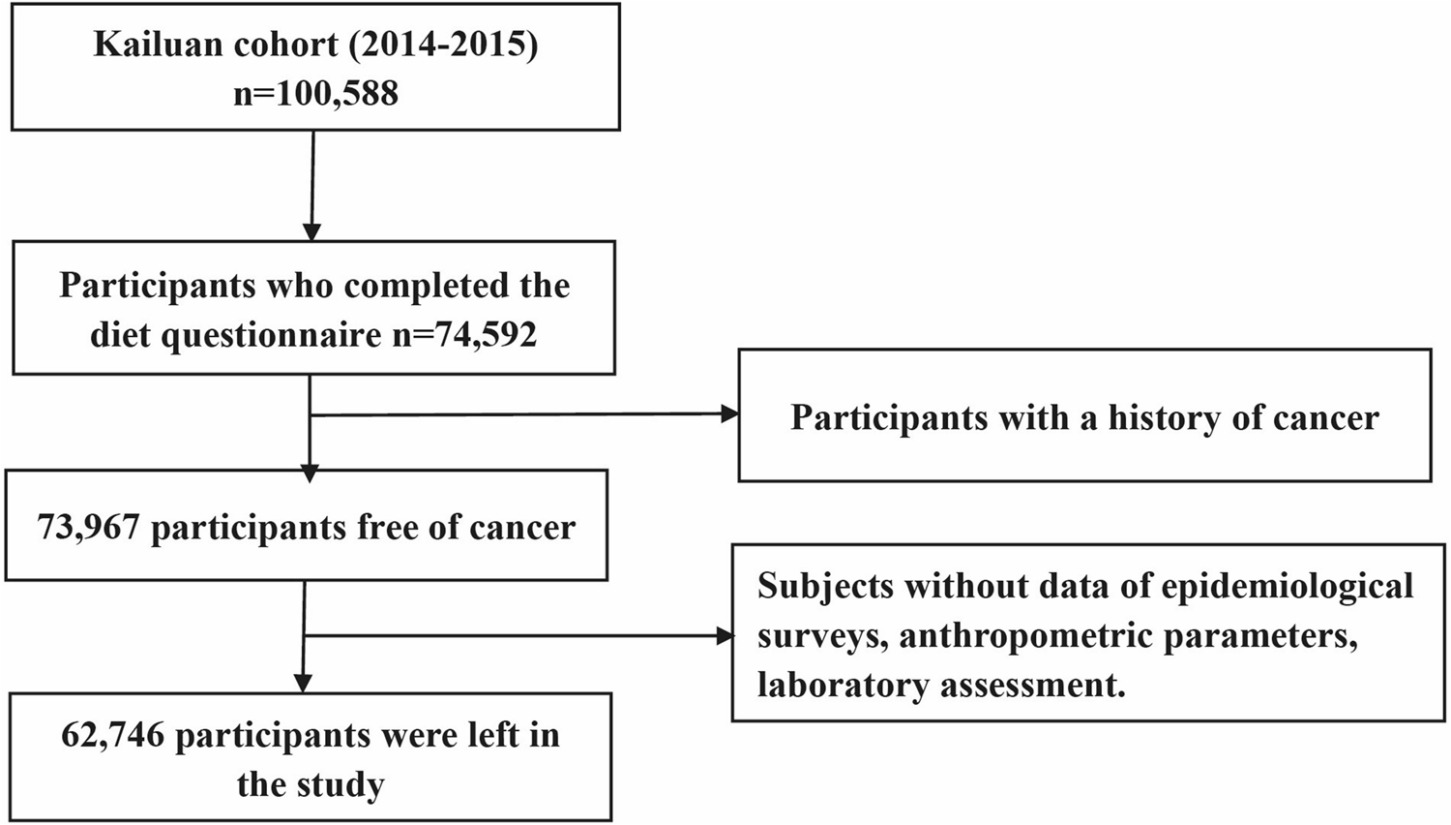
Flow chart of study participants

The rationale, study design, and methods of the Kailuan cohort study have previously been described.^15^ In short, the Kailuan study is a population-based prospective study based in the Kailuan community, which includes many employees of the Kailuan coal mining industry in Tangshan, Hebei Province in North China. From 2006 to 2007, 101,510 people (81,110 men and 20,400 women, aged 18–98 years) were included in the study and were followed up biennially. Each follow-up included face-to-face questionnaire surveys, body measurements, clinical examinations, and laboratory tests. In 2014, all individuals provided information on their habitual dietary intake with a semiquantitative Food Frequency Questionnaire (FFQ). Among the 74,592 participants who completed the FFQ, 625 were excluded due to a history of cancer, and 11,221 were excluded due to missing data on age, sex, body mass index (BMI), systolic blood pressure (SBP), diastolic blood pressure (DBP), fasting blood glucose (FBG) level, total cholesterol (TC) level, triglyceride (TG) level, CRP level, uric acid (UA) level, serum creatinine (Scr) level, occupation, family income, educational background, physical exercise, consumption of tobacco and alcoholic beverages, family history of cancer, sedentary lifestyle, tea consumption, high salt intake, high-fat diets, fatty liver, cirrhosis, gallstones, gallbladder polyps, hepatitis B surface antigen (HBsAg) level, hepatitis C virus (HCV) infection, and diet quality score. A total of 62,746 individuals were finally enrolled in this study, including 10,503 women and 52,243 men (Fig. 1).

### Assessment of Breakfast Frequency

The question “How many days do you generally consume breakfast in a typical week?” was used to assess breakfast frequency. There were four alternative responses: “no breakfast”, “1–2 times weekly”, “3–5 times weekly”, and “breakfast every day”. Participants were divided into four groups based on their responses to the question.

### Outcome Ascertainment

The following methods were used to identify incident GI cancer cases: (1) checking routine follow-up data for participants every 2 years until December 31, 2020, and (2) checking medical records from provincial vital statistics data, the Tangshan medical insurance system, and the Kailuan Social Security Information System once a year to obtain additional missing information. The Tangshan medical insurance system and the Kailuan social security system covered nearly all the participants’ health information. All cancer cases were reconfirmed based on either specific clinical features or positive histopathological results from hospitals where patients had received malignant tumor treatment. Potential cases were further assessed by two oncologists when pathological results were lacking. Only when two professionals made the same cancer diagnosis were cancer cases confirmed. GI cancers were documented as follows using the International Classification of Diseases, Tenth Revision (ICD-10): liver (C22); gallbladder or extrahepatic bile duct (C23-C24); gastric (C16); pancreatic (C25); small intestine (C17); esophageal (C15); and colorectal (C18-C21) cancers.

### Assessment of Covariates

The data of all covariates was collected in 2014. Information on age, sex, lifestyle (smoking status, alcohol consumption, physical activity, tea drinking, sedentary lifestyle, high-fat diet), sociodemographic data (education level, marital status, family income, occupation type), and personal and family medical histories (diabetes, hypertension, HCV infection, family history of cancer) was collected. Current alcohol usage was defined as drinking at least 100 mL of an alcoholic beverage each day for more than 6 months. Smoking was defined as having at least one cigarette per day for at least 6 months. Physical activity was defined as exercising at least 3 times per week, 30 min per session.

In 2014, dietary data were gathered using a validated semiquantitative FFQ. The semiquantitative FFQ comprises thirty-three food items and seven condiments and asks participants how frequently they consumed each food item in the previous year, with options of never, daily, weekly, or monthly. The FFQ was also used to calculate the amount of each meal consumed in liangs (50 g/liang). Adherence to the AHA recommendations (referred to as the “AHA diet score” in the current study) was used to assess diet quality, which was calculated based on the consumption of the following six components: fruits and vegetables, fish, Na, sweets, sugar-sweetened beverages, and whole grains, as previously described.^14^ The scale runs from 0 (worst) to 5 (best).

Weight and height were measured during the study visit, and BMI was calculated as weight (kg)/height (m^2^). After participants were sitting still, their SBP and DBP were recorded twice, and the average of the two readings was used in subsequent analysis. Hypertension was defined as an SBP ≥ 140 mmHg, a DBP ≥ 90 mmHg, a previous diagnosis, or the use of antihypertensive medication. An autoanalyzer (Hitachi 747; Hitachi) was used to measure FBG, TC, TG, CRP, SUA, Scr, and HBsAg levels. Diabetes was defined as an FBG level ≥ 7.0 mmol/L, a previous diagnosis, or the use of an oral hypoglycemic agent. Abdominal ultrasonography was used to detect liver cirrhosis, fatty liver, gallstone disease, and gallbladder polyps based on previously clinically established criteria^16,17^ or medical data from the Tangshan medical insurance system.

### Statistical Analysis

Continuous variables with normal and skewed distributions are presented as the mean ± standard deviation (SD) and median (interquartile range), and the comparisons were examined using the one-way analysis of variance (ANOVA) or the nonparametric Kruskal–Wallis test. Categorical variables were reported as absolute values with percentages and compared using the chi-squared test. Person-years were computed from the time of the baseline examination (2014–2015) until the date of the GI cancer diagnosis, death, or December 31, 2020, whichever occurred first. The proportional hazard assumption of breakfast skipping was evaluated by the Schoenfeld residual test. The Cox proportional hazards model was used to estimate the hazard ratios (HRs) and their 95% confidence intervals (CIs) for the association of breakfast skipping with the risk of GI cancer incidence.

Multivariate analyses included age (every 10 years), sex, BMI, TC level, TG level, Scr level, UA level, smoking status, drinking status, physical activity level, sedentary lifestyle, tea consumption, high-fat diet, diabetes, occupation type, family history of cancer, and diet quality score. In the pooled GI cancer studies, we only included the first reported cancer type. Site-specific studies were performed for all individuals with multiple relevant GI malignancies. In the site-specific analyses, multivariate analyses were further adjusted for liver cirrhosis, fatty liver, HBV infection, and HCV infection in the model of liver cancer; in addition, gallstone disease and gallbladder polyps were adjusted in the analyses of the gallbladder and biliary cancer. Subgroup analyses were performed for pooled GI cancers stratified by the median age (< 52 vs. ≥ 52 years), sex (men vs. women), and diet quality score (< 3 vs. ≥ 3). The interactions were further tested using multiplicative models.

Due to the close association of breakfast skipping with the risk of obesity, impaired glucose metabolism, and inflammation, we further explored whether the associations of breakfast consumption and GI cancer risk were mediated by obesity, inflammation, and insulin resistance, which were assessed by BMI, CRP level, and the TyG (fasting triglyceride-glucose) index (ln [TG (mmol/L) × FBG (mmol/L)]/2), respectively. The CAUSALMED procedure was used to perform the mediation analyses based on the variance–covariance matrix and the maximum likelihood method. This procedure calculated the total effect (the sum of the direct and indirect effects), the direct effect (the effect without the mediator’s influence), and the indirect effect (the effect of the independent variable on the outcome variable attributable to the mediator, which equals the effect of the independent variable on the mediator times the effect of the mediator on the outcome).

In the sensitivity analyses, we eliminated participants with cirrhosis and HCV infection at baseline due to the possible association of liver cirrhosis and HCV infection with GI cancer risk and reanalyzed the relationship to assess the robustness of our findings. To rule out the possibility of reverse causality, those who were diagnosed with cancer within the first year of follow-up were also eliminated.

## RESULTS

### Baseline Characteristics of the Study Population Stratified by Breakfast Frequency

The mean ± SD age for the study population was 51.30 ± 13.25 years (range: 18–98 years). Among the 62,746 participants in the current study, 53,796 (86%) reported breakfast consumption every day, and 5037 (8%) reported having no breakfast. Among the 4 prespecified groups, significant differences were found in the following: age; the levels of FBG, TC, TG, CRP, SUA, Scr, SBP, DBP, and BMI; and the percentages of men, educational levels, family income, family history of cancer, physical exercise, smoking status, drinking status, occupation type, sedentary lifestyle, tea drinking, high-fat diets, hypertension, diabetes mellitus, fatty liver, gallstone disease, gallbladder polyps, HBsAg seropositivity, and diet quality score. The prevalence of cirrhosis and HCV infection, on the other hand, did not differ significantly among the breakfast frequency groups (Table 1).

**Table 1:** Baseline characteristics of the participants stratified by breakfast consumption frequency

Variables	Breakfast everyday	3–5 breakfast/week	1–2 breakfast/week	No breakfast	*P*-value
*n *(%)	53,796	2,539	1,374	5,037	
Men (%)	44073 (81.93)	2236 (88.07)	1280 (93.16)	4654 (92.40)	
Age (year)	52.42 ± 13.94	44.41 ± 13.98	41.54 ± 12.63	45.52 ± 14.66	< 0.001
FBG (mmol/L)	5.85 ± 2.00	5.67 ± 1.78	5.69 ± 2.37	5.76 ± 1.72	< 0.001
TC (mmol/L)	5.17 ± 1.31	5.08 ± 2.01	5.05 ± 1.00	5.17 ± 1.58	< 0.001
BMI (Kg/m^2^)	24.93 ± 3.31	24.73 ± 3.33	24.72 ± 3.27	24.83 ± 3.41	< 0.001
SBP (mmHg)	136.02 ± 19.39	131.02 ± 17.48	130.87 ± 16.29	133.59 ± 17.95	< 0.001
DBP (mmHg)	82.30 ± 10.80	81.17 ± 10.49	80.94 ± 10.42	80.98 ± 10.69	< 0.001
TG (mmHg)	1.27 (0.87,1.93)	1.30 (0.88,2.03)	1.26 (0.87,2.08)	1.31 (0.92,1.99)	< 0.001
CRP (mg/L)	0.89 (0.38,1.90)	1.07 (0.47,2.10)	1.00 (0.43,2.00)	1.50 (0.80,2.30)	< 0.001
SUA (μmol/L)	320 (266,379)	332 (273,393)	338 (277,390)	322 (266,383)	< 0.001
Scr (μmol/L)	74.00 (64.00,86.30)	77.00 (67.0,90.60)	76.00 (68.00,85.00)	76.00 (68.00,85.00)	< 0.001
Per capita income (> 3000 ¥)	10307 (19.16)	545 (21.47)	284 (20.67)	791 (15.70)	< 0.001
Educational level (High school or above, %)	15719 (29.22)	1188 (46.79)	601 (43.74)	1702 (33.79)	< 0.001
Physical exercise (%)	3104 (7.36)	108 (4.25)	54 (3.93)	476 (9.54)	< 0.001
Smoking status (%)	23765 (44.18)	1337 (52.66)	772 (56.19)	1883 (37.38)	< 0.001
Drinking status (%)	25545 (47.48)	1472 (57.98)	796 (57.93)	1287 (25.55)	< 0.001
Family history of cancer (%)	1032 (1.92)	83 (3.27)	34 (2.47)	63 (1.25)	0.645
Diabetes mellitus (%)	6467 (12.02)	200 (7.88)	110 (8.01)	474 (9.41)	< 0.001
Hypertension (%)	21300 (39.59)	684 (26.94)	379 (27.58)	1698 (33.71)	< 0.001
Tea consumption (> 4 times/w, %)	1926 (3.58)	97 (3.82)	45 (3.28)	237 (4.70)	< 0.001
Sedentary lifestyle (> 8 h/d, %)	1382 (2.57)	62 (2.44)	22 (1.60)	155 (3.08)	0.016
High-fat diet (regularly, %)	2341 (4.35)	132 (5.20)	87 (6.33)	393 (7.80)	< 0.001
Fatty liver (%)	22205 (41.27)	1034 (40.72)	498 (36.24)	2357 (46.78)	< 0.001
Cirrhosis (%)	88 (0.16)	3 (0.12)	0 (0)	4 (0.08)	0.210
Gallstones (%)	949 (1.76)	35 (1.38)	10 (0.73)	88 (1.75)	0.016
Gallbladder polyp (%)	827 (1.54)	53 (2.09)	31 (2.26)	94 (1.87)	0.011
HCV infection (%)	50 (0.09)	1 (0.04)	1 (0.07)	4 (0.08)	0.829
HBsAg Seropositive (%)	824 (1.53)	24 (0.94)	19 (1.38)	56 (1.11)	0.013
Diet quality score	2.41 ± 1.12	2.50 ± 1.13	2.56 ± 1.14	1.84 ± 1.04	< 0.001
Occupation (labor worker, %)	30025 (55.89)	1164 (45.88)	667 (48.54)	3157 (62.71)	< 0.001

Note: *FBG* fasting blood glucose; *TC* total cholesterol; *BMI* body mass index; *SBP* systolic blood pressure; *DBP* diastolic blood pressure; *TG* triglyceride; *CRP* C-reactive protein; *SUA* serum uric acid; *Scr* serum creatinine

### The 1–2 Breakfasts/Week Group and No Breakfast Group Were Associated with an Elevated Risk of GI Cancer Incidence

During a median follow-up of 5.61 (5.18 ~ 6.08) years, 369 incident GI cancer cases were identified (esophageal cancer (*n* = 39), gastric cancer (*n* = 67), small-intestine cancer (*n* = 6), colorectal cancer (*n* = 136), liver cancer (*n* = 83), gallbladder or extrahepatic bile duct cancer (*n* = 15), pancreatic cancer (*n* = 26)); among them, 3 participants were diagnosed with multiple tumors. Results from the Schoenfeld residual test showed that the breakfast frequency met proportional hypothesis (*P* = 0.217). Compared with participants who had breakfast regularly, the 1–2 breakfasts/week group and no breakfast group were associated with a 2.35-fold (HR = 2.35, 95% CI: 1.28–4.32) and − 2.06-fold (HR = 2.06, 95% CI: 1.47–2.89) elevated risk of GI cancer incidence in the multivariate analyses (Table 2).

**Table 2:** The association of breakfast consumption frequency with the risk of GI cancers

Group	Cases/person-years	Crude models		Adjusted models	
		HR (95%CI)	*p*-value	HR (95%CI)	*p*-value
Breakfast everyday	303/297894	Ref.		Ref.	
3–5 breakfast/week	12/14290	0.82 (0.46,1.45)	0.487	1.23 (0.69,2.20)	0.485
1–2 breakfast/week	11/7879	1.35 (0.74,2.47)	0.328	**2.35 (1.28,4.32)**	0.006
No breakfast	44/28190	**1.53 (1.11,2.09)**	0.008	**2.06 (1.47,2.89)**	< 0.001
*P* for trend			0.036		< 0.001

Note: Adjusted models included age (every 10 years), sex, BMI, TC, TG, Scr, UA, smoking status, drinking status, physical activity, sedentary lifestyle, tea consumption, salt intake, high-fat diet, diabetes, occupation, family history of cancer and diet quality score. Results presented with bold values were statistically significant

Table 3 shows the association of breakfast consumption frequency with the risk of specific sites of GI cancers. In the site-specific analyses, after adjustments were made for the potential confounders, participants who consumed 1–2 breakfasts/week exhibited an increased risk of stomach cancer (HR = 3.45, 95% CI: 1.06–11.20) and liver cancer (HR = 3.42, 95% CI: 1.22–9.53). The no breakfast group was associated with an elevated risk of esophageal cancer (HR = 2.72, 95% CI: 1.05–7.03), colorectal cancer (HR = 2.32, 95% CI: 1.34–4.01), liver cancer (HR = 2.41, 95% CI: 1.23–4.71), and gallbladder and extrahepatic bile duct cancer (HR = 5.43, 95% CI: 1.34–21.93).

**Table 3:** The association of breakfast consumption frequency with the risk of specific sites of GI cancers

	Breakfast everyday	3–5 breakfast/week	1–2 breakfast/week	No breakfast
Esophageal cancer				
Cases/person-years	33/297894	0/14290	0/7879	6/28190
Adjusted HR (95%CI)	Ref.	NA	NA	**2.72 (1.05,7.03)**
Stomach cancer				
Cases/person-years	54/297894	4/14290	3/7879	7/28190
Adjusted HR (95%CI)	Ref.	2.24 (0.80,6.27)	**3.45 (1.06,11.20)**	2.02 (0.88,4.66)
Small intestine cancer				
Cases/person-years	6/297894	0/14290	0/7879	0/28190
Adjusted HR (95%CI)	Ref.	NA	NA	NA
Colorectal cancer				
Cases/person-years	111/297894	4/14290	4/7879	17/28190
Adjusted HR (95%CI)	Ref.	1.18 (0.43,3.20)	2.50 (0.91,6.85)	**2.32 (1.34,4.01)**
Liver cancer ^a^				
Cases/person-years	65/297894	2/14290	4/7879	12/28190
Adjusted HR (95%CI)	Ref.	1.05 (0.27,4.33)	**3.42 (1.22,9.53)**	**2.41 (1.23,4.71)**
Gallbladder and extrahepatic bile duct cancer ^b^				
Cases/person-years	11/297894	1/14290	0/7879	3/28190
Adjusted HR (95%CI)	Ref.	2.39 (0.30,18.99)	NA	**5.43 (1.34,21.93)**
Pancreatic cancer				
Cases/person-years	23/297894	1/14290	0/7879	2/28190
Adjusted HR (95%CI)	Ref.	1.66 (0,22,12.44)	NA	1.15 (0.25,5.26)

Note: Models were adjusted for age(every 10 years), sex, BMI, TC, TG, Scr, UA, smoking status, drinking status, physical activity, sedentary lifestyle, tea consumption, salt intake, high-fat diet, diabetes, occupation, family history of cancer and diet quality score

^a^Further adjusted for HBV infection, HCV infection, liver cirrhosis and fatty liver disease

^b^Further adjusted for gallstone disease and gallbladder polyp

Results presented with bold values were statistically significant

### Subgroup Analyses, Sensitivity Analyses, and Assessment of the Mediation Effect

**Fig. 2:**
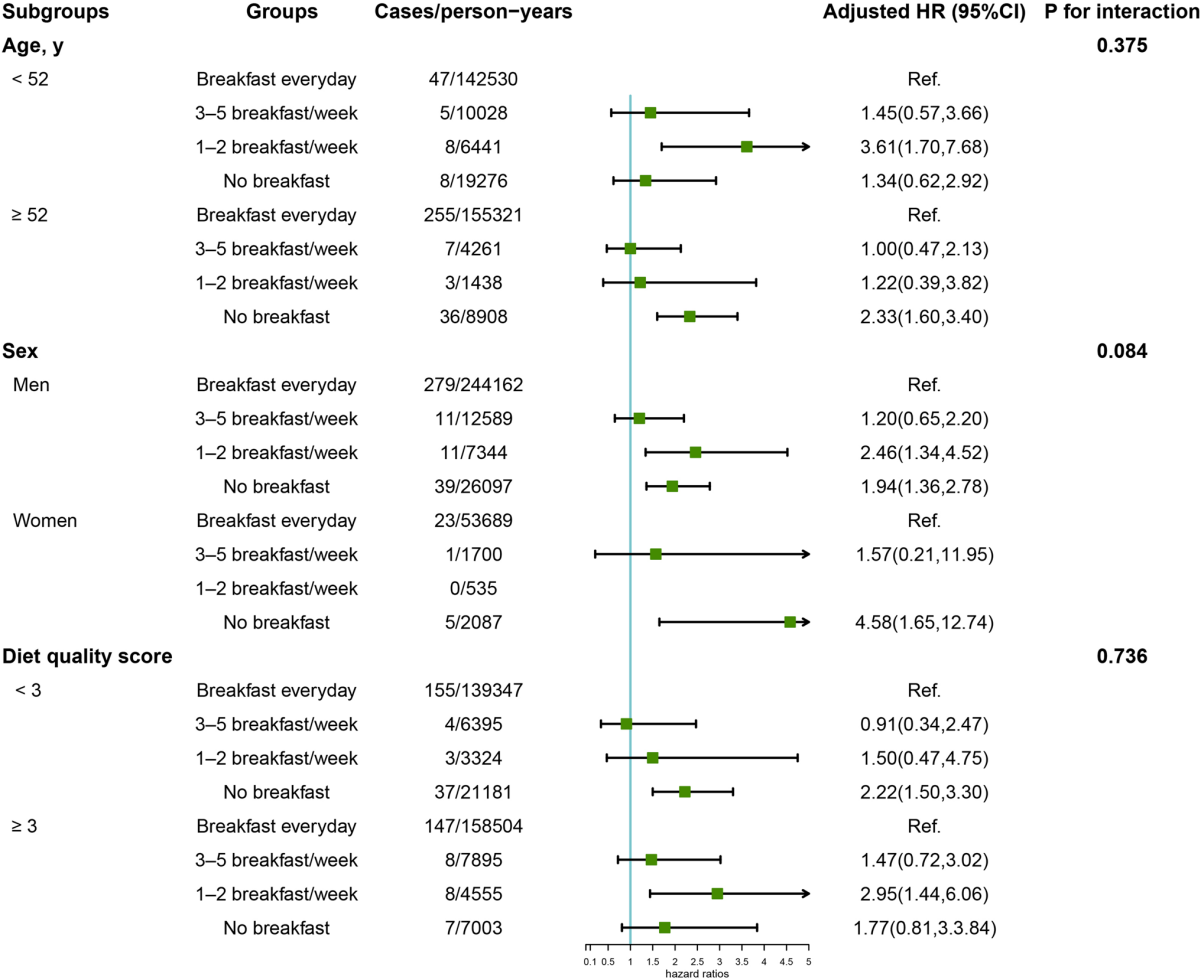
Subgroup analysis of the association between breakfast consumption and GI cancer risk

Figure 2 displays the effect of breakfast frequency on the occurrence of GI cancer after stratifying the participants by age, sex, and diet quality score. Significant associations of the 1–2 breakfasts/week or no breakfast groups with GI cancer risk were found in all subgroups. None of these variables significantly modified the associations between breakfast frequency and GI cancer risk (all *P* for interaction > 0.05). Sensitivity analyses did not substantially alter the conclusions but strengthened the HR to a higher level in some cancer sites. Notably, after excluding participants with GI cancers diagnosed within the 1^st^ year of follow-up, those who never ate breakfast also exhibited a higher risk of cancer; however, the risk of gallbladder and extrahepatic bile duct cancer was eliminated (Table 4). In the mediation effect analyses, the BMI, CRP, and TyG index levels did not mediate the association of breakfast frequency and the risk of GI cancer incidence (all *p* for mediation effect > 0.05, Supplementary Table 1).

**Table 4:** Sensitivity analyses of the association of breakfast consumption frequency with the risk of specific sites of GI cancers

	Breakfast everyday	3–5 breakfast/week	1–2 breakfast/week	No breakfast
Excluding GI cancer within 1^st^ year of follow-up (*n*=62710)				
Esophageal cancer	Ref.	NA	NA	**2.70 (1.04,6.99)**
Stomach cancer	Ref.	2.17 (0.66,7.10)	**4.31 (1.31,14.18)**	**2.68 (1.14,6.32)**
Small intestine cancer	Ref.	NA	NA	NA
Colorectal cancer	Ref.	1.29 (0.47,3.53)	2.70 (0.98,7.44)	**2.65 (1.52,4.62)**
Liver cancer ^a^	Ref.	1.35 (0.33,5.60)	**4.10 (1.45,11.59)**	**2.57 (1.23,5.38)**
Gallbladder and extrahepatic bile duct cancer ^b^	Ref.	2.36 (0.30,18.68)	NA	3.46 (0.68,17.55)
Pancreatic cancer	Ref.	1.90 (0,26,15.08)	NA	1.44 (0.31,6.73)
Excluding participants with cirrhosis (*n*=62665)				
Esophageal cancer	Ref.	NA	NA	**2.72 (1.05,7.03)**
Stomach cancer	Ref.	2.24 (0.80,6.27)	**3.43 (1.06,11.18)**	2.02 (0.88,4.65)
Small intestine cancer	Ref.	NA	NA	NA
Colorectal cancer	Ref.	1.18 (0.43,3.20)	2.49 (0.91,6.83)	**2.32 (1.34,4.00)**
Liver cancer ^a^	Ref.	1.12 (0.27,4.59)	**3.55 (1.27,9.91)**	**2.66 (1.36,5.22)**
Gallbladder and extrahepatic bile duct cancer ^b^	Ref.	2.46 (0.31,19.54)	NA	**5.48 (1.35,22.23)**
Pancreatic cancer	Ref.	1.66 (0,22,12.45)	NA	1.15 (0.25,5.26)
Excluding participants with HCV infection (*n*=62690)				
Esophageal cancer	Ref.	NA	NA	**2.72 (1.05,7.03)**
Stomach cancer	Ref.	2.24 (0.80,6.27)	**3.45 (1.06,11.20)**	2.02 (0.88,4.66)
Small intestine cancer	Ref.	NA	NA	NA
Colorectal cancer	Ref.	1.18 (0.43,3.20)	2.50 (0.91,6.85)	**2.31 (1.33,4.00)**
Liver cancer ^c^	Ref.	1.12 (0.27,4.59)	**3.40 (1.20,9.50)**	**2.40 (1.22,4.70)**
Gallbladder and extrahepatic bile duct cancer ^b^	Ref.	2.46 (0.31,19.54)	NA	**5.41 (1.32,21.91)**
Pancreatic cancer	Ref.	1.66 (0,22,12.45)	NA	1.15 (0.25,5.26)

Note: Models were adjusted for age (every 10 years), sex, BMI, TC, TG, Scr, UA, smoking status, drinking status, physical activity, sedentary lifestyle, tea consumption, salt intake, high-fat diet, diabetes, family history of cancer and diet quality score

^a^Further adjusted for HBV infection, HCV infection, liver cirrhosis, and fatty liver disease

^b^Further adjusted for gallstone disease and gallbladder polyp

^c^Further adjusted for HBV infection, liver cirrhosis, and fatty liver disease

Results presented with bold values were statistically significant

## DISCUSSION

In this large-scale prospective cohort study, we first discovered that participants who ate 1–2 breakfasts per week or had no breakfast had a greater risk of GI cancers, including esophageal, gastric, colorectal, liver, gallbladder, and extrahepatic bile duct cancer than those who ate breakfast every day. This association was unaffected by demographic, anthropometric, socioeconomic, or dietary factors. Subgroup analyses and sensitivity analyses further validated the robustness of the main findings.

In the current study, participants who skipped breakfast had an elevated risk of gastric cancer incidence, which is supported by several previous studies. A population-based case–control study involving 523 patients conducted in China found that breakfast skippers exhibited an elevated risk of gastric cancer.^18^ By analyzing data from 49 gastric cancer cases and 162 controls, Monserrat Verdalet-Olmedo et al. found that the skipping of breakfast elevated the risk of gastric cancer.^19^ In addition, the results from a cohort study of 34,128 men and 49,282 women showed that skipping breakfast was associated with an increased risk of mortality from cancer,^13^ which was supported by another prospective study conducted in the USA.^20^ However, a case–control study conducted in Mexico failed to find a positive association of breakfast skipping with the risk of gastric cancer.^21^

We also found that skipping breakfast was associated with elevated risks of esophageal, colorectal, liver, gallbladder, and extrahepatic bile duct cancers. Although much research has focused on the area of breakfast, no study thus far has examined the effect on the occurrence of cancer except for gastric cancer. The results from the cross-sectional MICOL found a positive association of an overnight fasting period of over 12 h with the risk of gallstones. According to previous studies, gallstone characteristics, such as the presence, size, and number, were associated with an increased risk of biliary tract cancer,^22^ liver cancer,^23^ and colorectal cancers.^24^

A large portion of the literature on the advantages of breakfast is concerned with health outcomes rather than nutritional results. Several large prospective studies have found a link between breakfast intake and a decreased risk of obesity and weight increase.^9,25^ The results from several prospective studies, including the Health Professionals Follow-Up Study, the Nurses’ Health Study, a Japanese study and the German EPIC cohort study, found a positive association between skipping breakfast and impaired glucose metabolism.^11,26–28^ In addition, several epidemiological studies have reported an inverse association of breakfast consumption with cardiovascular disease (CVD) risk^29,30^ as well as its related risk factors.^11^ Data from the Spanish PESA (Progression of Early Subclinical Atherosclerosis) cohort emphasized the hypothesis that omitting breakfast was related to increased odds of atherosclerosis, regardless of the presence of traditional cardiovascular risk factors.^31^ In brief, breakfast intake was related to a decrease in cardiometabolic risk factors.

In the current study, participants who ate breakfast 1–2 times a week had the highest risk of stomach cancer and liver cancer, which can be explained by the relatively short follow-up period. We further assumed that the participants maintained regular eating habits for a long time and applied dietary data from the 2014–2015 examination to the population who participated in the 2012–2013 healthy examination. A significant association of breakfast frequency with GI cancer risk was observed only in participants who did not have breakfast (Supplementary Table S2). This extended follow-up method, however, changed the prospective design to a retrospective design and may lead to the misclassification of the participants.

Several underlying mechanisms may help explain the association between breakfast skipping and subsequent GI cancer risk. First, habitually skipping breakfast was associated with elevated concentrations of CRP.^14^ Long-term low-grade inflammation promotes tumor development and progression via (1) oxidation^32^; (2) mutation, DNA methylation and posttranslational variations caused by inflammatory response mediators, including cytokines, free radicals, prostaglandins and growth factors^32^; and (3) the activation of the NF-κB pathway, which is essential for the invasion of cancer.^33^ Second, breakfast consumption lowers the risk of obesity and metabolic risk factors. The roles of obesity and metabolic disturbance in cancer etiopathogenesis comprise^34^ the following: (1) hyperinsulinemia/insulin resistance and abnormalities of the insulin-like growth factor-I (IGF-I) system and signaling; (2) alterations in adipocytokine pathophysiology; (3) an altered intestinal microbiome; and (4) microenvironment and cellular perturbations. Third, compared with regular breakfast eaters,^35^ habitual breakfast skippers are usually associated with unhealthy lifestyle behaviors, such as smoking and physical inactivity, which are independent risk factors for the development of cancer. However, the association of breakfast consumption and GI cancer risk remained after adjusting for these lifestyle factors. Notably, in the current study, breakfast skippers were relatively younger, with a lower prevalence of smoking, drinking, and physical inactivity.

A major strength of this current study is that it provided a unique perspective on the possible effects of breakfast consumption on the occurrence of incident GI cancer risk based on a population-based cohort study. Furthermore, this study investigated a wide range of potentially confounding characteristics, such as lifestyle behaviors and a history of cancer-related disorders. The prospective research design and large sample size are further strengths of this study.

Limitations in the current study should also be noted. First, breakfast consumption frequency was collected via self-report questionnaires, which may have led to the misclassification of the participants. Second, the current study included more men than women due to the industrial character of the Kailuan Company. However, the sample size of women was still high enough to investigate the association. Third, different morning meals and patterns may have varying effects on cancer risk. However, we did not collect information on the exact meals eaten during breakfast. Fourth, *Helicobacter pylori* infection has been reported to be closely associated with chronic gastritis, peptic ulcer disease, and gastric cancer, all of which can cause upper gastrointestinal symptoms and affect lifestyle habits, including breakfast consumption. However, the lacked data on *Helicobacter pylori* infection hinders us from assessing the absolute risk of breakfast consumption more precisely. Fifth, short median follow-up (5.61 years) and relatively young age may not be sufficient to detect a true relationship between breakfast and GI cancer risk. However, due to the small sample size of the no breakfast group, subgroup analyses stratified by age or sex were yielded to null associations in the current study (data not shown). Future studies with a larger sample size and longer follow-up should be conducted to validate the robustness of the main findings in the current study. Sixth, endoscopy is the golden method of screening for gastrointestinal cancers, especially asymptomatic occult cancers. However, due to the cost-effectiveness policy of the Kailuan cohort, no endoscopy was performed on the individuals. Finally, calorie restriction (CR) is beneficial for longevity,^36^ and skipping breakfast is also a form of CR. In fact, skipping breakfast is currently mainly due to staying up later, then, getting up later. In addition, staying up late is a common major risk factor for non-communicable diseases (NCDs), including cardiovascular disease (CVD), diabetes, and cancer.^37–39^ Ultimately, an unhealthy lifestyle alters the biological rhythm or circadian clock, which then affects the human immune system.^39^ Unfortunately, the Kailuan study did not collect the information on the overall calorie intake and sleep rhythm. Future studies need to be conducted to explore the relationship between breakfast skipping and overall calorie intake, as well as the impact of breakfast skipping on the human immunity.

## CONCLUSIONS

In this large-scale cohort, we found that habitually skipping breakfast was associated with a greater risk of GI cancers, including esophageal, gastric, colorectal, liver, gallbladder, and extrahepatic bile duct cancers. This study should be a stepping stone to further research on breakfast consumption and GI cancer risk. Future research should better elucidate the potential mechanisms of breakfast skipping for carcinogenesis. Importantly, specific prevention efforts could be focused on the population with a habit of skipping breakfast.

## Supplementary Information

Below is the link to the electronic supplementary material.

Supplementary file1 (DOCX 21.5 KB)

## Data Availability

Data will be made available upon reasonable request.

## Abbreviations

BMI: Body mass index
CIs: Confidence intervals
CRP: C-reactive protein
CVD: Cardiovascular disease
DBP: Diastolic blood pressure
FBG: Fasting blood glucose
FFQ: Food Frequency Questionnaire
GI: Gastrointestinal
HBsAg: Hepatitis B surface antigen
HCV: Hepatitis C virus
HRs: Hazard ratios
SBP: Systolic blood pressure
Scr: Serum creatinine
TC: Total cholesterol
TG: Triglyceride
TyG: Fasting triglyceride-glucose
UA: Uric acid
