# Supplementary material for: Habitually Skipping Breakfast Is Associated with the Risk of Gastrointestinal Cancers: Evidence from the Kailuan Cohort Study
Source: J Gen Intern Med. 2023 Mar 3;38(11):2527–36. doi: 10.1007/s11606-023-08094-7 (PMC10465444; doi:10.1007/s11606-023-08094-7)
Supplement: Supplementary file 1 — Supplementary file1 (DOCX 21.5 KB) [file 11606_2023_8094_MOESM1_ESM.docx]

**Supplementary Tables**

**Table S1. The association of breakfast consumption frequency with the risk of specific sites of GI cancers from 2012 to 2020**

|  | **Breakfast everyday**  **n=48807** | **3–5 breakfast/week**  **n=2151** | **1–2 breakfast/week**  **n=1131** | **No breakfast**  **n=4460** |
| --- | --- | --- | --- | --- |
| **Esophageal cancer** |  |  |  |  |
| Cases/person-years | 36/372880 | 0/16647 | 2/8840 | 7/33555 |
| Adjusted HR (95%CI) | Ref. | NA | 3.66(0.86,15.59) | **2.98(1.30,6.86)** |
| **Stomach cancer** |  |  |  |  |
| Cases/person-years | 90/372880 | 5/16647 | 2/8840 | 11/33555 |
| Adjusted HR (95%CI) | Ref. | 2.15(0.86,5.39) | 1.80(0.44,7.39) | **2.14(1.09,4.19)** |
| **Small intestine cancer** |  |  |  |  |
| Cases/person-years | 11/372880 | 0/16647 | 0/8840 | 0/33555 |
| Adjusted HR (95%CI) | Ref. | NA | NA | NA |
| **Colorectal cancer** |  |  |  |  |
| Cases/person-years | 141/372880 | 4/16647 | 4/8840 | 20/33555 |
| Adjusted HR (95%CI) | Ref. | 0.82(0.26,2.59) | 2.17(0.80,5.94) | **2.66(1.64,4.31)** |
| **Liver cancer ^a^** |  |  |  |  |
| Cases/person-years | 97/372880 | 0/16647 | 3/8840 | 13/33555 |
| Adjusted HR (95%CI) | Ref. | NA | 2.43(0.76,7.80) | **1.90(0.94,3.82)** |
| **Gallbladder and extrahepatic bile duct cancer ^b^** |  |  |  |  |
| Cases/person-years | 8/372880 | 0/16647 | 0/8840 | 4/33555 |
| Adjusted HR (95%CI) | Ref. | NA | NA | **9.30(4.87,32.42)** |
| **Pancreatic cancer** |  |  |  |  |
| Cases/person-years | 23/372880 | 1/16647 | 0/8840 | 3/33555 |
| Adjusted HR (95%CI) | Ref. | 1.85(0,24,14.07) | NA | 2.52(0.72,8.81) |

**Note: Models were adjusted for age (every 10 years), sex, BMI, TC, TG, Scr, UA, smoking status, drinking status, physical activity, sedentary lifestyle, tea consumption, salt intake, high-fat diet, diabetes, occupation, family history of cancer and diet quality score (2014).**

**a: Further adjusted for HBV infection, liver cirrhosis and fatty liver disease.**

**b: Further adjusted for gallstone disease and gallbladder polyp.**

**Table S2. Significance of mediation effect by CRP, BMI, and TyG index in the Association of BMI and WC with CRC risk**

|  | **Total effect** | **Natural direct**  **effect** | **Natural indirect**  **effect** | **Proportion mediated** |
| --- | --- | --- | --- | --- |
| **CRP** |  |  |  |  |
| **Esophageal cancer** | 0.027 | 0.024 | 0.933 | 0.933 |
| **Stomach cancer** | 0.046 | 0.049 | 0.830 | 0.830 |
| **Small intestine cancer** | 0.509 | 0.470 | 0.474 | 0.627 |
| **Colorectal cancer** | 0.002 | 0.001 | 0.340 | 0.361 |
| **Liver cancer ^a^** | 0.001 | 0.001 | 0.687 | 0.689 |
| **Gallbladder and extrahepatic bile duct cancer ^b^** | 0.044 | 0.043 | 0.476 | 0.504 |
| **Pancreatic cancer** | 0.677 | 0.705 | 0.686 | 0.772 |
| **BMI** |  |  |  |  |
| **Esophageal cancer** | 0.027 | 0.024 | 0.153 | 0.290 |
| **Stomach cancer** | 0.046 | 0.046 | 0.934 | 0.935 |
| **Small intestine cancer** | 0.509 | 0.515 | 0.462 | 0.623 |
| **Colorectal cancer** | 0.002 | 0.002 | 0.649 | 0.653 |
| **Liver cancer ^a^** | 0.001 | 0.001 | 0.423 | 0.437 |
| **Gallbladder and extrahepatic bile duct cancer ^b^** | 0.044 | 0.042 | 0.155 | 0.253 |
| **Pancreatic cancer** | 0.677 | 0.680 | 0.752 | 0.801 |
| **TyG index** |  |  |  |  |
| **Esophageal cancer** | 0.027 | 0.026 | 0.899 | 0.900 |
| **Stomach cancer** | 0.046 | 0.047 | 0.509 | 0.531 |
| **Small intestine cancer** | 0.509 | 0.504 | 0.523 | 0.646 |
| **Colorectal cancer** | 0.002 | 0.002 | 0.238 | 0.270 |
| **Liver cancer ^a^** | 0.001 | 0.001 | 0.933 | 0.933 |
| **Gallbladder and extrahepatic bile duct cancer ^b^** | 0.044 | 0.042 | 0.181 | 0.274 |
| **Pancreatic cancer** | 0.677 | 0.665 | 0.174 | 0.691 |

**Note: Models were adjusted for age (every 10 years), sex, BMI, TC, TG, Scr, UA, smoking status, drinking status, physical activity, sedentary lifestyle, tea consumption, salt intake, high-fat diet, diabetes, occupation, family history of cancer and diet quality score (2014).**

**a: Further adjusted for HBV infection, liver cirrhosis and fatty liver disease.**

**b: Further adjusted for gallstone disease and gallbladder polyp.**
